# Association of the 24-hour movement behaviours composition with workers’ chronic musculoskeletal pain

**DOI:** 10.1371/journal.pone.0346414

**Published:** 2026-04-03

**Authors:** Aino Kitayama, Yu-Tai Liu, Ai Shibata, Sayaka Kurosawa, Koichiro Oka

**Affiliations:** 1 Graduate School of Sport Sciences, Waseda University, Tokorozawa, Saitama, Japan; 2 Institute of Health and Sport Sciences, University of Tsukuba, Tsukuba, Ibaraki, Japan; 3 College of Sport and Wellness, Rikkyo University, Niiza, Saitama, Japan; 4 Faculty of Sport Sciences, Waseda University, Tokorozawa, Saitama, Japan; University of Illinois Urbana-Champaign, UNITED STATES OF AMERICA

## Abstract

**Background:**

Chronic musculoskeletal pain is a significant symptom among workers. 24-hour movement behaviours comprising sleep, sedentary behaviour, light-intensity physical activity, and moderate-to-vigorous-intensity physical activity are associated factors. However, the relationships between these behaviours and workers’ chronic musculoskeletal pain, considering the interrelationship between the behaviours, are still unclear. This study aimed to investigate the associations of 24-hour movement behaviours with workers’ low-back and neck/shoulder pain.

**Methods:**

In 2023, cross-sectional survey was conducted targeting adults who registered for a Japanese Internet survey system. Time for 24-hour movement behaviours and other demographic characteristics (age, gender, marital status, education, household income, height, weight, smoking, alcohol, chronic disease, work hours, and job activity) were self-reported. The intensity of low-back and neck/shoulder pain was evaluated using the numerical rating scale and a score of ≥4 was considered as moderate-to-severe pain. Compositional logistic regression and isotemporal substitution were performed to examine the associations of 24-hour movement behaviours and time reallocations between the behaviours with moderate-to-severe low-back and neck/shoulder pain, adjusted for all the demographic variables.

**Results:**

1,665 full-time workers (women: 35.8%, mean age: 42.1 ± 10.2 years) were analysed. Increased sleep and decreased light-intensity or moderate-to-vigorous-intensity physical activity were associated with lower odds of moderate-to-severe low-back (adjusted odds ratio [AOR] = 0.54, 95% confidence interval [CI] = 0.40–0.72; 1.45, 1.25–1.69; 1.17, 1.06–1.30, respectively) and neck/shoulder pain (AOR = 0.60, 95% CI = 0.45–0.80; 1.37, 1.19–1.60; 1.12, 1.01–1.24, respectively). Reallocating sleep from the other behaviours was associated with decreased probabilities of low-back and neck/shoulder pain, whereas replacing sedentary behaviour or light-intensity physical activity with more intense activity was associated with increased probabilities. However, the results of moderate-to-vigorous-intensity physical activity reallocation were no longer significant when restricted to complete cases.

**Conclusions:**

Considering the interrelationship of 24-hour movement behaviours, sleep was favourably associated with workers’ low-back and neck/shoulder pain.

## Introduction

Low-back, neck, and shoulder pain are the most prevalent chronic musculoskeletal pain symptoms worldwide. According to the Global Burden of Disease Study 2021, approximately 629 million and 206 million people suffered low-back and neck pain, respectively [1]. Also, a systematic review reported that global prevalence of shoulder pain is a median of 16.0% (ranging 0.67–55.2%) for various reference periods [2]. Particularly, workers are a key population at risk of these chronic musculoskeletal pain symptoms due to their unchanged awkward posture and psychosocial stress while at work [3,4]. Chronic musculoskeletal pain including low-back and neck/shoulder pain has a potential to lead to physical limitation [5], increased risk of chronic diseases such as cardiovascular disease and cancer [6], and reduced quality of life [7] as well as presenteeism and absenteeism in working environment [8]. Therefore, managing workers’ chronic musculoskeletal pain conditions is one of the most critical issues to be addressed in public and occupational health [9].

To date, epidemiological studies have shown that physical activity, sedentary behaviour, and sleep, collectively known as 24-hour movement behaviours, are correlated lifestyle factors of chronic musculoskeletal pain for adults. Specifically, both short and long sleep [10,11], longer total and workplace sedentary time [12], and low levels of moderate-to-vigorous-intensity physical activity [13,14] were associated with chronic musculoskeletal pain including low-back and neck/shoulder pain. Despite growing evidence on the relationships between these 24-hour movement behaviours and chronic musculoskeletal pain, the behaviours were independently examined in previous studies, thereby failing to consider their interrelationship within 24 hours; increasing time for one behaviour results in decreasing time for the other behaviours. Thus, further research is needed to examine the associations between 24-hour movement behaviours and chronic musculoskeletal pain.

One of the possible and appropriate approaches to account for this interdependence is the compositional data analysis (CoDA), which has been utilized in recent studies investigating the relationships of 24-hour movement behaviours with various health outcomes allowing to consider the co-dependency of 24-hour movement behaviours [15]. One previous study applied CoDA to examine the relationships between 24-hour movement behaviours and low-back pain in adults and older adults [16]. They found reallocating sleep from any other behaviours was favourably associated with intensity and frequency of low-back pain, while reallocating moderate-to-vigorous-intensity physical activity was unfavourably associated with low-back pain outcomes. Also, replacing sedentary behaviour with light-intensity physical activity was associated with lower intensity of low-back pain. However, time reallocations were conducted for only low-back pain sufferers in this previous study, and the results might not be applied to working population. Moreover, there was a discrepancy of reference periods between behavioural (the past week) and pain variables (the past year). This mismatch might limit the interpretation of the study findings because it enabled participants who once had low-back pain to cure them of pain during a week prior to the study participation. In addition, no studies have investigated the relationships between 24-hour movement behaviours and neck/shoulder pain using CoDA, even though this type of pain is also significant.

Therefore, the present study aimed to explore the association between 24-hour movement behaviours with low-back and neck/shoulder pain in full-time workers using CoDA approach.

## Methods

### Participants and data collection

This study included cross-sectional data taken from a nationwide online survey in 2023. This survey was conducted through a Japanese internet research company (MyVoice Communication, Inc. Tokyo, Japan), where over one million individuals voluntarily registered to participate in online surveys. Between 24th and 28th February 2023, an invitation e-mail with a survey link was sent to 19,081 registered individuals aged 20–59 years, who were randomly selected stratified by gender and age group (the 20s, 30s, 40s, and 50s) from the database. As a result, a total of 3,000 adults, including 1,500 of each gender and 750 of each age group, responded to this survey (response rate = 15.7%) and received an incentive of exchangeable points at allied facilities. Among the respondents, only full-time workers (n = 1,840) who were employed by company or self-employed were eligible for the study purpose. Moreover, those who reported 0 minutes for sleep (n = 10) or sedentary behaviour (n = 22), whose calculated time for light-intensity physical activity was 0 minutes or less (n = 141), and whose highest educational attainment was unclear (n = 2) were excluded from the analyses. All participants were requested to read the survey information presented on the first page of the survey, which had been reviewed and approved by the Ethics Committee, and provided electronic informed consent by clicking “I agree to participate”. When they clicked “I disagree to participate”, they were allowed to quit the survey. This survey was completely voluntary and participants were entitled to withdraw at any time without detriment. This study was approved by the Institutional Ethics Committee of Waseda University (2022−407).

### Measures

#### 24-hour movement behaviours.

Time spent on each 24-hour movement behaviour was assessed using a brief 24-hour movement behaviours’ questionnaire consisting of four items (i.e., one item for each behaviour) [17]. Participants reported average daily hours and minutes for sleep, sedentary behaviour, and light-intensity and moderate-to-vigorous-intensity physical activity for a typical week. This instrument has previously shown acceptable validity (rho = 0.39–0.60) and reliability (ICC = 0.72–0.90) [17]. Complying with the process in which this questionnaire was developed, time spent in light-intensity physical activity was calculated by subtracting the sum of durations of the other behaviours from 24 hours when the sum of reported durations of all the behaviours did not equal 24 hours.

#### Low-back and neck/shoulder pain.

Pain intensity was estimated using the numerical rating scale (NRS). In this assessment, participants reported the intensity of low-back and neck/shoulder pain within the last month by choosing the most suitable number from 0 to 10, with 0 representing ‘not at all’ and 10 representing ‘the worst possible pain’. This scale has previously shown excellent validity and reliability among patients with rheumatic and other chronic pain conditions [18] and reported the lower incorrect response rates compared with other pain scales including the visual analogue scale, verbal rating scale, and faces pain scale [19]. A score of 4 or more, typically classified as moderate-to-severe pain, is often used to identify individuals whose function may be interfered [20]. In order to interpret the results of this study meaningfully towards those stakeholders, participants reporting from 0 to 3 and 4–10 were classified into “none-to-mild” and “moderate-to-severe” categories, respectively.

#### Other variables.

Participants reported age, gender (men/women), marital status (married/unmarried, widowed, or divorced), highest education (university or higher/2-year college/junior high school or high school), and household income (<5,000,000 yen/5,000,000- <10,000,000 yen/ ≥10,000,000 yen) as socio-demographic information. The collected health-related information included height, weight, smoking status (current smoker/non-smoker), alcohol consumption (≥5 days per week/1–4 days per week/ <1 day per week), and history of at least one chronic disease on the following lists (yes/no): cerebrovascular disease, cardiovascular disease, cancers, diabetes, hypertension, hypercholesterolemia, gout, and depression. Body mass index (kg/m^2^) was calculated from self-reported height (m) and weight (kg). Also, they provided information on hours of work per day and job activity (sitting/standing/walking/physical).

### Statistical analysis

Firstly, descriptive analyses were conducted for socio-demographic, health-related, and work-related variables as well as musculoskeletal pain variables shown as means and standard deviations for continuous data, and frequencies and percentages for categorical data. Then, this study applied CoDA to examine the associations of each 24-hour movement behaviour with moderate-to-severe low-back and neck/shoulder pain. Each 24-hour movement behaviour variable was transformed into isometric log ratio coordinates (ilrs). For example, a set of three ilrs for moderate-to-vigorous-intensity physical activity was calculated using the following formula: ilr_MVPA1_$=\sqrt{\frac{3}{4}}ln\frac{\text{MVPA}}{\sqrt[{3}]{sleep•SB•LPA}}$, ilr_MVPA2_$=\sqrt{\frac{2}{3}}ln\frac{\text{LPA}}{\sqrt{sleep•SB}}$, ilr_MVPA3_$=\sqrt{\frac{1}{2}}ln\frac{\text{SB}}{sleep}$. For each behaviour, the first coordinate out of the set of three ilrs was used as independent variables in the multivariate binary logistic regression model, adjusted for all the socio-demographic, health-related, and work-related variables. Similar to the previous study [16], the 0 values for moderate-to-vigorous-intensity physical activity were imputed using the log-ratio expectation-maximisation algorithm for 698 participants, where detection of the potential imputed values was limited to 1 minute and the imputation was performed using maximum likelihood estimation based on multiplicative simple replacement. In addition, compositional isotemporal substitution analyses were performed to calculate predicted probabilities of moderate-to-severe low-back and neck/shoulder pain by reallocating 10, 20, and 30 minutes to each 24-hour movement behaviour from the other behaviours. Durations of reallocations were selected based on the compositional mean of moderate-to-vigorous-intensity physical activity (approximately 38 minutes) in the current study, where more than 38 minutes of reallocation would not be feasible. The changes in the probability of moderate-to-severe low-back and neck/shoulder pain by time reallocations were reported. Descriptive and compositional analyses were conducted in IBM SPSS Statistics 30.0 (SPSS Inc., IBM Japan, Ltd, Tokyo, Japan) and R version 4.4.0 (R Foundation for Statistical Computing, Vienna, Austria) with “tidyverse”, “compositions”, “zCompositions”, “robCompositions”, “broom”, “effectsize”, and “boot” packages, respectively. A significant p-value was set at less than 5%.

#### Sensitivity analysis.

Two sensitivity analyses were conducted to check if the current analysis was robust. Firstly, about 40% of the participants reported 0 minutes for moderate-to-vigorous-intensity physical activity and their data was imputed in the analysis. Therefore, the first sensitivity analysis used the remaining participants with complete data (n = 967). Secondly, some studies have used a different threshold (NRS score of 5) for pain outcomes to detect participants with moderate-to-severe pain [20]. Thus, the second sensitivity analysis used 5 in NRS for the threshold.

## Results

A total of 1,665 full-time workers were included in the analyses. Table 1 shows participants’ socio-demographic, health-related, and work-related characteristics. Overall, mean (± SD) age and BMI were 42.1 (± 10.2) years and 22.5 (± 3.7) kg/m^2^, respectively. Majority were men and unmarried, and completed university education, while about one-third of participants earned less than 5 million yen annually. Around 20 percent of participants were smokers, drank alcohol five or more days a week, and had at least one chronic disease. They worked, on average, 10.5 hours in a day and more than two-thirds had sitting-based jobs. About one out of three participants had moderate-to-severe low-back and/or neck/shoulder pain.

**Table 1:** Characteristics of the study participants (n = 1,665).

		Mean ± SD or n (%)
**Age**, years		42.1 ± 10.2
**Gender**	Men	1069 (64.2)
	Women	596 (35.8)
**Marital status**	Married	818 (49.1)
	Unmarried/Widowed/Divorced	847 (50.9)
**Education**	University or higher	1101 (66.1)
	2-year college	270 (16.2)
	Junior high school or High school	294 (17.7)
**Household income**	<5,000,000 yen	607 (36.5)
	5,000,000 to <10,000,000 yen	778 (46.7)
	≥10,000,000 yen	280 (16.8)
**BMI**, kg/m^2^		22.5 ± 3.7
**Smoking**	Current smoker	366 (22.0)
	Non-smoker	1299 (78.0)
**Alcohol**	<1 day per week	888 (53.3)
	1 to 4 days per week	415 (24.9)
	≥5 days per week	362 (21.7)
**Chronic diseases**	Yes	346 (20.8)
	No	1319 (79.2)
**Hours of work per day**		10.5 ± 9.5
**Job activity**	Sitting	1149 (69.0)
	Standing	220 (13.2)
	Walking	237 (14.2)
	Physical	59 (3.5)
**Low-back pain**	Moderate-to-severe	584 (35.1)
	None-to-mild	1081 (64.9)
**Neck/shoulder pain**	Moderate-to-severe	645 (38.7)
	None-to-mild	1020 (61.3)

Abbreviation: BMI = body mass index, SD = standard deviation.

Mean composition of 24-hour movement behaviours and its associations with low-back and neck/shoulder pain are presented in Table 2. The compositional means of sleep, sedentary behaviour, and light-intensity and moderate-to-vigorous-intensity physical activity were 7.86 hours, 7.10 hours, 8.40 hours and 0.64 hours, respectively. More time for sleep was associated with lower odds of both low-back and neck/shoulder pain, whereas more time for light-intensity and moderate-to-vigorous-intensity physical activity was associated with higher odds of low-back and neck/shoulder pain. There was no association between sedentary behaviour within the 24-hour composition and low-back and neck/shoulder pain.

**Table 2:** Compositional means of 24-hour movement behaviours and their associations with low-back and neck/shoulder pain.

	Compositional mean (h)	Low-back pain		Neck/shoulder pain	
		AOR (95%CI) ^a^	p-value	AOR (95%CI) ^a^	p-value
Sleep	7.86	0.54 (0.40-0.72)	<0.001	0.60 (0.45-0.80)	<0.001
SB	7.10	1.10 (0.93-1.29)	0.274	1.08 (0.92-1.27)	0.931
LPA	8.40	1.45 (1.25-1.69)	<0.001	1.37 (1.19-1.60)	<0.001
MVPA	0.64	1.17 (1.06-1.30)	0.003	1.12 (1.01-1.24)	0.026

^a^Adjusted for age, gender, marital status, education, household income, BMI, smoking, alcohol, chronic diseases, hours of work, and job activity.

Abbreviation: AOR = adjusted odds ratio, BMI = body mass index, CI = confidence interval, h = hour, LPA = light-intensity physical activity, MVPA = moderate-to-vigorous-intensity physical activity, SB = sedentary behaviour.

Relationships between substituting time spent in one behaviour for another behaviour with the rest of two behaviours fixed and low-back and neck/shoulder pain are shown in Tables 3 and 4. Replacing sleep with the other behaviours, sedentary behaviour with physical activity in any kind, and light-intensity physical activity with moderate-to-vigorous-intensity physical activity were associated with increased probability of moderate-to-severe low-back and neck/shoulder pain, with the exception of reallocation of time between light-intensity and moderate-to-vigorous-intensity physical activity for neck/shoulder pain. On the other hand, replacing sedentary behaviour with sleep, light-intensity physical activity with sleep or sedentary behaviour, and moderate-to-vigorous-intensity physical activity with the other behaviours were associated with decreased probability of moderate-to-severe low-back and neck/shoulder pain.

**Table 3:** Differences in predicted probabilities of low-back pain when reallocating time between 24-hour movement behaviours.

Changes (min)	To	Difference (95%CI) ^a^	To	Difference (95%CI) ^a^	To	Difference (95%CI) ^a^
Reallocation from sleep…						
10	SB	0.0026 (0.0010 to 0.0041) *	LPA	0.0034 (0.0020 to 0.0048) *	MVPA	0.0084 (0.0041 to 0.0126) *
20		0.0052 (0.0021 to 0.0082) *		0.0069 (0.0041 to 0.0097) *		0.0157 (0.0082 to 0.0233) *
30		0.0079 (0.0033 to 0.0125) *		0.0104 (0.0063 to 0.0146) *		0.0225 (0.0119 to 0.0331) *
Reallocation from SB…						
10	Sleep	−0.0025 (−0.0041 to −0.0009) *	LPA	0.0008 (0.0003 to 0.0014) *	MVPA	0.0057 (0.0019 to 0.0096) *
20		−0.0050 (−0.0080 to −0.0019) *		0.0017 (0.0006 to 0.0027) *		0.0104 (0.0036 to 0.0172) *
30		−0.0074 (−0.0120 to −0.0027) *		0.0025 (0.0008 to 0.0041) *		0.0143 (0.0043 to 0.0242) *
Reallocation from LPA…						
10	Sleep	−0.0034 (−0.0048 to −0.0020) *	SB	−0.0009 (−0.0014 to −0.0003) *	MVPA	0.0049 (0.0011 to 0.0086) *
20		−0.0067 (−0.0095 to −0.0039) *		−0.0018 (−0.0029 to −0.0007) *		0.0086 (0.0015 to 0.0156) *
30		−0.0100 (−0.0141 to −0.0059) *		−0.0027 (−0.0043 to −0.0011) *		0.0115 (0.0022 to 0.0209) *
Reallocation from MVPA…						
10	Sleep	−0.0099 (−0.0152 to −0.0046) *	SB	−0.0075 (−0.0125 to −0.0024) *	LPA	−0.0066 (−0.0115 to −0.0018) *
20		−0.0228 (−0.0358 to −0.0098) *		−0.0181 (−0.0305 to −0.0057) *		−0.0165 (−0.0284 to −0.0047) *
30		−0.0433 (−0.0691 to −0.0176) *		−0.0368 (−0.0614 to −0.0122) *		−0.0345 (−0.0597 to −0.0093) *

*p < 0.05.

a Adjusted for age, gender, marital status, education, household income, BMI, smoking, alcohol, chronic diseases, hours of work, and job activity.

Abbreviation: BMI = body mass index, CI = confidence interval, LPA = light-intensity physical activity, min = minute, MVPA = moderate-to-vigorous-intensity physical activity, SB = sedentary behaviour.

**Table 4:** Differences in predicted probabilities of neck/shoulder pain when reallocating time between 24-hour movement behaviours.

Changes (min)	To	Difference (95%CI) ^a^	To	Difference (95%CI) ^a^	To	Difference (95%CI) ^a^
Reallocation from sleep…						
10	SB	0.0024 (0.0007 to 0.0041) *	LPA	0.0032 (0.0016 to 0.0048) *	MVPA	0.0070 (0.0025 to 0.0115) *
20		0.0048 (0.0013 to 0.0082) *		0.0064 (0.0033 to 0.0095) *		0.0132 (0.0048 to 0.0216) *
30		0.0072 (0.0022 to 0.0123) *		0.0097 (0.0052 to 0.0143) *		0.0188 (0.0072 to 0.0305) *
Reallocation from SB…						
10	Sleep	−0.0023 (−0.0041 to −0.0006) *	LPA	0.0008 (0.0002 to 0.0014) *	MVPA	0.0046 (0.0006 to 0.0086) *
20		−0.0046 (−0.0080 to −0.0012) *		0.0016 (0.0004 to 0.0028) *		0.0083 (0.0008 to 0.0159) *
30		−0.0068 (−0.0119 to −0.0017) *		0.0024 (0.0006 to 0.0041) *		0.0114 (0.0011 to 0.0216) *
Reallocation from LPA…						
10	Sleep	−0.0032 (−0.0046 to −0.0017) *	SB	−0.0009 (−0.0014 to −0.0003) *	MVPA	0.0038 (−0.0004 to 0.0079)
20		−0.0063 (−0.0093 to −0.0033) *		−0.0017 (−0.0029 to −0.0006) *		0.0066 (−0.0011 to 0.0143)
30		−0.0094 (−0.0139 to −0.0049) *		−0.0027 (−0.0044 to −0.0009) *		0.0088 (−0.0019 to 0.0194)
Reallocation from MVPA…						
10	Sleep	−0.0084 (−0.0139 to −0.0028) *	SB	−0.0061 (−0.0115 to −0.0006) *	LPA	−0.0052 (−0.0104 to −0.0001) *
20		−0.0193 (−0.0328 to −0.0058) *		−0.0148 (−0.0275 to −0.0022) *		−0.0132 (−0.0262 to −0.0002) *
30		−0.0369 (−0.0655 to −0.0083) *		−0.0305 (−0.0571 to −0.0039) *		−0.0282 (−0.0563 to −0.0000) *

*p < 0.05.

^a^Adjusted for age, gender, marital status, education, household income, BMI, smoking, alcohol, chronic diseases, hours of work, and job activity.

Abbreviation: BMI = body mass index, CI = confidence interval, LPA = light-intensity physical activity, min = minute, MVPA = moderate-to-vigorous-intensity physical activity, SB = sedentary behaviour.

### Sensitivity analysis

The results of the first sensitivity analysis are presented in S1–S3 Tables. After excluding those with 0 minutes for moderate-to-vigorous-intensity physical activity, compositional means of sedentary behaviour and light-intensity physical activity decreased while that of moderate-to-vigorous-intensity physical activity increased. Also, similar trends were observed for the associations of each 24-hour movement behaviour with low-back and neck/shoulder pain although the associations of moderate-to-vigorous-intensity physical activity with the pain outcomes were no longer statistically significant. The results of changes in probabilities of low-back and neck/shoulder pain by isotemporal substitution show similar trends for the reallocations from and to sleep and between sedentary behaviour and light-intensity physical activity, whereas the results for the reallocation from moderate-to-vigorous-intensity physical activity to sedentary behaviour or light-intensity physical activity and the opposite reallocations were statistically insignificant.

The results of the second sensitivity analysis are shown in S4–S6 Tables. Using the NRS score of 5 as a threshold, similar results were observed for the associations of 24-hour movement behaviours with low-back and neck/shoulder pain outcomes. Also, similar trends remained for the changes in predicted probabilities of the outcomes by the time reallocations between these behaviours with only slight differences in effect size.

## Discussion

This study investigated the associations of 24-hour movement behaviours and reallocations of time between the behaviours with low-back and neck/shoulder pain in full-time workers. Less sleep and more light-intensity and moderate-to-vigorous-intensity physical activity within 24 hours were associated with higher odds of low-back and neck/shoulder pain. Moreover, reallocating sleep from any other behaviours was associated with decreased probabilities of low-back and neck/shoulder pain, whereas reallocating moderate-to-vigorous-intensity physical activity from any other behaviours or light-intensity physical activity from sleep or sedentary behaviour was associated with increased probabilities of both musculoskeletal pain. Time reallocations in the opposite direction resulted in the opposite associations.

Sleep, both as a component of 24-hour movement behaviours and a target of time reallocation from the other behaviours, was favourably associated with low-back and neck/shoulder pain. Similar trends were found in previous studies regardless of CoDA use, where more time spent in sleep and reallocating sleep from the other behaviours were associated with lower odds of chronic spinal pain such as low-back pain [16,21,22]. These results suggest that increasing time for sleep could be an effective approach to manage chronic musculoskeletal pain. The finding of this study also supported the recommendation for the daily sleep duration to prevent diseases in 24-hour movement behaviours’ guidelines for adults (i.e., 7–9 hours) [23].

Both light-intensity and moderate-to-vigorous-intensity physical activity were unfavourably associated with low-back and neck/shoulder pain. These findings disagreed with previous studies without CoDA use reporting that light-intensity physical activity was not associated with musculoskeletal disability in older adults [24] and exercise or leisure time physical activity was favourably associated with low-back and neck pain in office workers [25], occupational drivers [26], and patients with non-specific low-back pain [13]. However, as similar results to the present study were observed in a previous study using CoDA [16], the potential benefit of increasing moderate-to-vigorous-intensity physical activity on chronic musculoskeletal pain might be affected by diminishing the other behaviours especially sleep. Another possible explanation might be the domain-specific relationships of physical activity with chronic musculoskeletal pain. To date, physical activity paradox has been gradually suggested to consider its domain for the association with health outcomes [27]. Recent studies found physical activity at work was associated with increased pain, while leisure physical activity with decreased pain [28,29]. Hence, moderate-to-vigorous-intensity physical activity assessed in this and the previous studies might mainly be accumulated while at work. However, since information on domains of the behaviours were unavailable, further studies clarifying each physical activity’s domain would be needed to identify the domain-specific relationships with chronic musculoskeletal pain.

This study found no significant association between sedentary behaviour and chronic musculoskeletal pain, which was inconsistent with the most recent systematic reviews without CoDA approach arguing there was a significant relationship between sedentary behaviour and low-back pain [12,30]. Therefore, the relationship of sedentary behaviour with chronic musculoskeletal pain might be diminished after CoDA application, implying the unfavourable association of sedentary behaviour was possibly brought by a compensation of decreased sleep. Also, time reallocation of sedentary behaviour from sleep led to unfavourable results, whereas sedentary behaviour reallocation from moderate-to-vigorous-intensity physical activity showed favourable associations with low-back and neck/shoulder pain in accordance with previous study using CoDA [16]. Together with the previous study, the result of the current study suggests that it could be plausible increasing sleep with decreasing sedentary time to manage chronic musculoskeletal pain. In contrast, the result of reallocating sedentary behaviour from light-intensity physical activity did not match the finding in the previous study reporting that replacement of sedentary behaviour with light-intensity physical activity was associated with lower odds of low-back pain intensity among adults and older adults with low-back pain [16]. This inconsistency might be caused by the difference of analysed samples between the current and previous studies. Some previous studies showed that certain light-intensity physical activities including prolonged standing or static postures were associated with workers’ low-back discomfort [31] and neck/shoulder complaints [32]. Thus, increasing light-intensity physical activity along with decreasing sedentary behaviour might not bring favourable results in working population. Moreover, workers might have replaced sedentary behaviour with light-intensity physical activity (e.g., gentle stretching) to relieve their pain in this study. However, this causal inference could not be concluded due to the cross-sectional design.

The key strength of the present study was use of CoDA to investigate associations of 24-hour movement behaviours with chronic musculoskeletal pain. Nevertheless, there are several limitations to be acknowledged. To begin with, 24-hour movement behaviours were assessed using a self-reported tool, which can cause recall bias [33]. Even though this tool showed acceptable validity and reliability, there were gaps from the accelerometry assessment [17]. Moreover, this tool captured total durations of each behaviour and, thus, there was no information about domains in which each activity occurred. Even though moderate-to-vigorous-intensity physical activity might be accumulated at work considering the results of the current study, it could not be examined due to lack of information. Future studies might collect domain-specific information on each behaviour and explore the distinct associations by the activity domains. Additionally, 40% of the participants reported 0 minutes for moderate-to-vigorous-intensity physical activity using this tool. Thus, zero-imputation for this value might cause modelling error. The sensitivity analysis without those with 0 minutes for moderate-to-vigorous-intensity physical activity resulted in similar trends, however, there was a noticeable difference in the associations of moderate-to-vigorous-intensity physical activity with pain outcomes. Therefore, the cautious interpretation of the results might be needed. Next, it was unclear whether the participants reported the intensity on only chronic low-back and neck/shoulder pain. Even though they were asked the pain intensity over the past month and assumed to likely report on their chronic pain, they possibly reported on acute pain, which is typically caused by injury. This mismatch might affect the current results. Furthermore, this study may be limited by a lack of information on stress and comorbidities or other conditions that could influence musculoskeletal pain (e.g., paralysis). This study did not include these variable due to unavailability of the data. However, stress had a strong relationship with chronic pain [34] and comorbidities may affect pain perception [35], therefore, further studies would be needed using these variables in investigating the relationships between 24-hour movement behaviours and chronic musculoskeletal pain. Finally, the cross-sectional design of this study limited interpretation of findings; causal relationships between 24-hour movement behaviours and chronic musculoskeletal pain could not be concluded. The relationships between 24-hour movement behaviours and chronic musculoskeletal pain are bidirectional [36,37]; movement behaviours or sleep can affect chronic musculoskeletal pain status, while chronic musculoskeletal pain can also affect movement behaviours or sleep level. Further studies using longitudinal and interventional designs would be needed to explore longitudinal and causal relationships.

## Conclusions

24-hour movement behavioural composition was cross-sectionally associated with workers’ low-back and neck/shoulder pain. Reallocating sleep from the other behaviours was favourably associated with these chronic musculoskeletal pain. Sedentary behaviour and light-intensity physical activity was unfavourably associated with workers’ low-back and neck/shoulder pain when replaced with more intense behaviours. The results related to moderate-to-vigorous-intensity physical activity were no longer statistically significant using the complete case analysis and, therefore, should be interpreted cautiously. Further longitudinal or interventional studies might be needed to clarify the longitudinal or causal relationships between these behaviours and chronic musculoskeletal pain.

## Supporting information

S1 Table A sensitivity analysis for compositional means of 24-hour movement behaviours and their associations with low-back and neck/shoulder pain with complete cases (n = 967). (DOCX)

S2 Table A sensitivity analysis for differences in predicted probabilities of low-back pain when reallocating time between 24-hour movement behaviours with complete cases (n = 967). (DOCX)

S3 Table A sensitivity analysis for differences in predicted probabilities of neck/shoulder pain when reallocating time between 24-hour movement behaviours with complete cases (n = 967). (DOCX)

S4 Table A sensitivity analysis for compositional means of 24-hour movement behaviours and their associations with low-back and neck/shoulder pain detected by NRS of ≥5 (n = 1,665). (DOCX)

S5 Table A sensitivity analysis for differences in predicted probabilities of low-back pain detected by NRS of ≥5 when reallocating time between 24-hour movement behaviours (n = 1,665). (DOCX)

S6 Table A sensitivity analysis for differences in predicted probabilities of neck/shoulder pain detected by NRS of ≥5 when reallocating time between 24-hour movement behaviours (n = 1,665). (DOCX)
