# Supplementary material for: Association of the 24-hour movement behaviours composition with workers’ chronic musculoskeletal pain
Source: PLoS One. 2026 Apr 3;21(4):e0346414. doi: 10.1371/journal.pone.0346414 (PMC13048427; doi:10.1371/journal.pone.0346414)
Supplement: S1 Table — (DOCX) [file pone.0346414.s001.docx]

S1 Table. A sensitivity analysis for compositional means of 24-hour movement behaviours and their associations with low-back and neck/shoulder pain with complete cases (n=967).

|  | Compositional mean (h) | Low-back pain | | Neck/shoulder pain | |
| --- | --- | --- | --- | --- | --- |
|  |  | AOR (95%CI) ^a^ | p-value | AOR (95%CI) ^a^ | p-value |
| Sleep | 7.83 | 0.55 (0.38-0.78) | <0.001 | 0.61 (0.43-0.86) | <0.001 |
| SB | 6.67 | 1.11 (0.90-1.38) | 0.316 | 1.06 (0.86-1.31) | 0.581 |
| LPA | 8.06 | 1.43 (1.20-1.73) | <0.001 | 1.38 (1.16-1.65) | <0.001 |
| MVPA | 1.45 | 1.14 (0.96-1.36) | 0.133 | 1.12 (0.95-1.33) | 0.190 |

^a^ Adjusted for age, gender, marital status, education, household income, BMI, smoking, alcohol, chronic diseases, hours of work, and job activity

Abbreviation: AOR = adjusted odds ratio, BMI = body mass index, CI = confidence interval, h = hour, LPA = light-intensity physical activity, MVPA = moderate-to-vigorous-intensity physical activity, SB = sedentary behaviour
