# Supplementary material for: Association of the 24-hour movement behaviours composition with workers’ chronic musculoskeletal pain
Source: PLoS One. 2026 Apr 3;21(4):e0346414. doi: 10.1371/journal.pone.0346414 (PMC13048427; doi:10.1371/journal.pone.0346414)
Supplement: S2 Table — (DOCX) [file pone.0346414.s002.docx]

S2 Table. A sensitivity analysis for differences in predicted probabilities of low-back pain when reallocating time between 24-hour movement behaviours with complete cases (n=967).

| Changes (min) | To | Difference (95%CI) ^a^ | To | Difference (95%CI) ^a^ | To | Difference (95%CI) ^a^ |
| --- | --- | --- | --- | --- | --- | --- |
| Reallocation from sleep… | |  |  |  |  |  |
| 10 | SB | 0.0027 (0.0007 to 0.0048) * | LPA | 0.0036 (0.0018 to 0.0054) * | MVPA | 0.0048 (0.0010 to 0.0087) * |
| 20 |  | 0.0055 (0.0013 to 0.0098) * |  | 0.0072 (0.0035 to 0.0109) * |  | 0.0095 (0.0021 to 0.0169) * |
| 30 |  | 0.0084 (0.0021 to 0.0147) * |  | 0.0109 (0.0057 to 0.0161) * |  | 0.0141 (0.0033 to 0.0248) * |
| Reallocation from SB… | |  |  |  |  |  |
| 10 | Sleep | -0.0027 (-0.0048 to -0.0006) * | LPA | 0.0008 (0.0000 to 0.0016) * | MVPA | 0.0021 (-0.0014 to 0.0056) |
| 20 |  | -0.0053 (-0.0095 to -0.0012) * |  | 0.0016 (-0.0000 to 0.0032) |  | 0.0039 (-0.0024 to 0.0102) |
| 30 |  | -0.0079 (-0.0140 to -0.0018) * |  | 0.0023 (-0.0002 to 0.0049) |  | 0.0055 (-0.0039 to 0.0148) |
| Reallocation from LPA… | |  |  |  |  |  |
| 10 | Sleep | -0.0035 (-0.0054 to -0.0017) * | SB | -0.0009 (-0.0017 to -0.0001) * | MVPA | 0.0012 (-0.0022 to 0.0046) |
| 20 |  | -0.0070 (-0.0107 to -0.0033) * |  | -0.0017 (-0.0034 to -0.0001) * |  | 0.0022 (-0.0041 to 0.0084) |
| 30 |  | -0.0105 (-0.0161 to -0.0048) * |  | -0.0027 (-0.0051 to -0.0003) * |  | 0.0029 (-0.0061 to 0.0118) |
| Reallocation from MVPA… | |  |  |  |  |  |
| 10 | Sleep | -0.0050 (-0.0092 to -0.0009) * | SB | -0.0024 (-0.0060 to 0.0012) | LPA | -0.0016 (-0.0052 to 0.0021) |
| 20 |  | -0.0104 (-0.0191 to -0.0017) * |  | -0.0052 (-0.0127 to 0.0024) |  | -0.0035 (-0.0115 to 0.0044) |
| 30 |  | -0.0161 (-0.0304 to -0.0018) * |  | -0.0085 (-0.0209 to 0.0040) |  | -0.0060 (-0.0190 to 0.0070) |

*p<0.05

^a^ Adjusted for age, gender, marital status, education, household income, BMI, smoking, alcohol, chronic diseases, hours of work, and job activity

Abbreviation: BMI = body mass index, CI = confidence interval, LPA = light-intensity physical activity, min = minute, MVPA = moderate-to-vigorous-intensity physical activity, SB = sedentary behaviour
