# Supplementary material for: Association of the 24-hour movement behaviours composition with workers’ chronic musculoskeletal pain
Source: PLoS One. 2026 Apr 3;21(4):e0346414. doi: 10.1371/journal.pone.0346414 (PMC13048427; doi:10.1371/journal.pone.0346414)
Supplement: S3 Table — (DOCX) [file pone.0346414.s003.docx]

S3 Table. A sensitivity analysis for differences in predicted probabilities of neck/shoulder pain when reallocating time between 24-hour movement behaviours with complete cases (n=967).

| Changes (min) | To | Difference (95%CI) ^a^ | To | Difference (95%CI) ^a^ | To | Difference (95%CI) ^a^ |
| --- | --- | --- | --- | --- | --- | --- |
| Reallocation from sleep… | |  |  |  |  |  |
| 10 | SB | 0.0023 (0.0002 to 0.0045) * | LPA | 0.0033 (0.0014 to 0.0052) * | MVPA | 0.0044 (0.0004 to 0.0085) * |
| 20 |  | 0.0047 (0.0002 to 0.0091) * |  | 0.0066 (0.0029 to 0.0104) * |  | 0.0087 (0.0010 to 0.0165) * |
| 30 |  | 0.0071 (0.0007 to 0.0135) * |  | 0.0100 (0.0043 to 0.0158) * |  | 0.0129 (0.0019 to 0.0239) * |
| Reallocation from SB… | |  |  |  |  |  |
| 10 | Sleep | -0.0023 (-0.0045 to -0.0001) * | LPA | 0.0010 (0.0002 to 0.0018) * | MVPA | 0.0021 (-0.0014 to 0.0056) |
| 20 |  | -0.0045 (-0.0088 to -0.0002) * |  | 0.0019 (0.0002 to 0.0037) * |  | 0.0040 (-0.0024 to 0.0104) |
| 30 |  | -0.0067 (-0.0131 to -0.0003) * |  | 0.0028 (0.0003 to 0.0054) * |  | 0.0057 (-0.0034 to 0.0147) |
| Reallocation from LPA… | |  |  |  |  |  |
| 10 | Sleep | -0.0033 (-0.0052 to -0.0014) * | SB | -0.0010 (-0.0018 to -0.0002) * | MVPA | 0.0011 (-0.0024 to 0.0046) |
| 20 |  | -0.0065 (-0.0103 to -0.0026) * |  | -0.0020 (-0.0037 to -0.0004) * |  | 0.0020 (-0.0046 to 0.0085) |
| 30 |  | -0.0097 (-0.0156 to -0.0039) * |  | -0.0031 (-0.0057 to -0.0006) * |  | 0.0026 (-0.0069 to 0.0120) |
| Reallocation from MVPA… | |  |  |  |  |  |
| 10 | Sleep | -0.0047 (-0.0089 to -0.0004) * | SB | -0.0024 (-0.0063 to 0.0015) | LPA | -0.0014 (-0.0052 to 0.0023) |
| 20 |  | -0.0096 (-0.0190 to -0.0002) * |  | -0.0052 (-0.0133 to 0.0030) |  | -0.0032 (-0.0113 to 0.0048) |
| 30 |  | -0.0150 (-0.0300 to -0.0000) * |  | -0.0084 (-0.0215 to 0.0046) |  | -0.0056 (-0.0189 to 0.0077) |

*p<0.05

^a^ Adjusted for age, gender, marital status, education, household income, BMI, smoking, alcohol, chronic diseases, hours of work, and job activity

Abbreviation: BMI = body mass index, CI = confidence interval, LPA = light-intensity physical activity, min = minute, MVPA = moderate-to-vigorous-intensity physical activity, SB = sedentary behaviour
