# Supplementary material for: Association of the 24-hour movement behaviours composition with workers’ chronic musculoskeletal pain
Source: PLoS One. 2026 Apr 3;21(4):e0346414. doi: 10.1371/journal.pone.0346414 (PMC13048427; doi:10.1371/journal.pone.0346414)
Supplement: S4 Table — (DOCX) [file pone.0346414.s004.docx]

S4 Table. A sensitivity analysis for compositional means of 24-hour movement behaviours and their associations with low-back and neck/shoulder pain detected by NRS of ≥5 (n=1,665).

|  | Compositional mean (h) | Low-back pain | | Neck/shoulder pain | |
| --- | --- | --- | --- | --- | --- |
|  |  | AOR (95%CI) ^a^ | p-value | AOR (95%CI) ^a^ | p-value |
| Sleep | 7.86 | 0.55 (0.40-0.73) | <0.001 | 0.60 (0.45-0.81) | <0.001 |
| SB | 7.10 | 1.07 (0.91-1.28) | 0.404 | 1.03 (0.87-1.22) | 0.722 |
| LPA | 8.40 | 1.41 (1.21-1.66) | <0.001 | 1.40 (1.20-1.64) | <0.001 |
| MVPA | 0.64 | 1.21 (1.09-1.35) | <0.001 | 1.15 (1.03-1.28) | 0.010 |

^a^ Adjusted for age, gender, marital status, education, household income, BMI, smoking, alcohol, chronic diseases, hours of work, and job activity

Abbreviation: AOR = adjusted odds ratio, BMI = body mass index, CI = confidence interval, h = hour, LPA = light-intensity physical activity, MVPA = moderate-to-vigorous-intensity physical activity, SB = sedentary behaviour
