# Supplementary material for: Association of the 24-hour movement behaviours composition with workers’ chronic musculoskeletal pain
Source: PLoS One. 2026 Apr 3;21(4):e0346414. doi: 10.1371/journal.pone.0346414 (PMC13048427; doi:10.1371/journal.pone.0346414)
Supplement: S5 Table — (DOCX) [file pone.0346414.s005.docx]

S5 Table. A sensitivity analysis for differences in predicted probabilities of low-back pain detected by NRS of ≥5 when reallocating time between 24-hour movement behaviours (n=1,665).

| Changes (min) | To | Difference (95%CI) ^a^ | To | Difference (95%CI) ^a^ | To | Difference (95%CI) ^a^ |
| --- | --- | --- | --- | --- | --- | --- |
| Reallocation from sleep… | |  |  |  |  |  |
| 10 | SB | 0.0020 (0.0007 to 0.0033) * | LPA | 0.0027 (0.0015 to 0.0039) * | MVPA | 0.0079 (0.0041 to 0.0117) * |
| 20 |  | 0.0041 (0.0013 to 0.0068) * |  | 0.0055 (0.0030 to 0.0080) * |  | 0.0149 (0.0081 to 0.0217) * |
| 30 |  | 0.0062 (0.0022 to 0.0102) * |  | 0.0083 (0.0046 to 0.0120) * |  | 0.0213 (0.0119 to 0.0307) * |
| Reallocation from SB… | |  |  |  |  |  |
| 10 | Sleep | -0.0020 (-0.0033 to -0.0006) * | LPA | 0.0007 (0.0002 to 0.0012) * | MVPA | 0.0058 (0.0024 to 0.0093) * |
| 20 |  | -0.0039 (-0.0065 to -0.0012) * |  | 0.0014 (0.0004 to 0.0023) * |  | 0.0106 (0.0046 to 0.0167) * |
| 30 |  | -0.0058 (-0.0099 to -0.0017) * |  | 0.0020 (0.0006 to 0.0035) * |  | 0.0147 (0.0067 to 0.0227) * |
| Reallocation from LPA… | |  |  |  |  |  |
| 10 | Sleep | -0.0027 (-0.0039 to -0.0015) * | SB | -0.0007 (-0.0012 to -0.0002) * | MVPA | 0.0051 (0.0018 to 0.0084) * |
| 20 |  | -0.0053 (-0.0078 to -0.0028) * |  | -0.0015 (-0.0024 to -0.0005) * |  | 0.0092 (0.0030 to 0.0153) * |
| 30 |  | -0.0079 (-0.0115 to -0.0042) * |  | -0.0022 (-0.0037 to -0.0007) * |  | 0.0124 (0.0043 to 0.0205) * |
| Reallocation from MVPA… | |  |  |  |  |  |
| 10 | Sleep | -0.0094 (-0.0140 to -0.0047) * | SB | -0.0075 (-0.0118 to -0.0032) * | LPA | -0.0068 (-0.0111 to -0.0026) * |
| 20 |  | -0.0216 (-0.0328 to -0.0104) * |  | -0.0180 (-0.0284 to -0.0076) * |  | -0.0167 (-0.0271 to -0.0063) * |
| 30 |  | -0.0408 (-0.0633 to -0.0183) * |  | -0.0359 (-0.0569 to -0.0150) * |  | -0.0342 (-0.0558 to -0.0125) * |

*p<0.05

^a^ Adjusted for age, gender, marital status, education, household income, BMI, smoking, alcohol, chronic diseases, hours of work, and job activity

Abbreviation: BMI = body mass index, CI = confidence interval, LPA = light-intensity physical activity, min = minute, MVPA = moderate-to-vigorous-intensity physical activity, SB = sedentary behaviour
