# Supplementary material for: Association of the 24-hour movement behaviours composition with workers’ chronic musculoskeletal pain
Source: PLoS One. 2026 Apr 3;21(4):e0346414. doi: 10.1371/journal.pone.0346414 (PMC13048427; doi:10.1371/journal.pone.0346414)
Supplement: S6 Table — (DOCX) [file pone.0346414.s006.docx]

S6 Table. A sensitivity analysis for differences in predicted probabilities of neck/shoulder pain detected by NRS of ≥5 when reallocating time between 24-hour movement behaviours (n=1,665).

| Changes (min) | To | Difference (95%CI) ^a^ | To | Difference (95%CI) ^a^ | To | Difference (95%CI) ^a^ |
| --- | --- | --- | --- | --- | --- | --- |
| Reallocation from sleep… | |  |  |  |  |  |
| 10 | SB | 0.0019 (0.0004 to 0.0034) * | LPA | 0.0029 (0.0015 to 0.0042) * | MVPA | 0.0071 (0.0029 to 0.0112) * |
| 20 |  | 0.0038 (0.0008 to 0.0069) * |  | 0.0057 (0.0030 to 0.0085) * |  | 0.0133 (0.0056 to 0.0210) * |
| 30 |  | 0.0058 (0.0013 to 0.0103) * |  | 0.0087 (0.0044 to 0.0129) * |  | 0.0189 (0.0082 to 0.0297) * |
| Reallocation from SB… | |  |  |  |  |  |
| 10 | Sleep | -0.0018 (-0.0034 to -0.0003) * | LPA | 0.0010 (0.0004 to 0.0015) * | MVPA | 0.0052 (0.0014 to 0.0089) * |
| 20 |  | -0.0036 (-0.0066 to -0.0007) * |  | 0.0019 (0.0008 to 0.0030) * |  | 0.0094 (0.0025 to 0.0162) * |
| 30 |  | -0.0054 (-0.0101 to -0.0007) * |  | 0.0028 (0.0012 to 0.0044) * |  | 0.0129 (0.0036 to 0.0222) * |
| Reallocation from LPA… | |  |  |  |  |  |
| 10 | Sleep | -0.0028 (-0.0041 to -0.0015) * | SB | -0.0010 (-0.0015 to -0.0005) * | MVPA | 0.0042 (0.0006 to 0.0078) * |
| 20 |  | -0.0056 (-0.0083 to -0.0029) * |  | -0.0020 (-0.0030 to -0.0009) * |  | 0.0073 (0.0007 to 0.0139) * |
| 30 |  | -0.0083 (-0.0122 to -0.0045) * |  | -0.0030 (-0.0046 to -0.0014) * |  | 0.0098 (0.0004 to 0.0193) * |
| Reallocation from MVPA… | |  |  |  |  |  |
| 10 | Sleep | -0.0085 (-0.0136 to -0.0033) * | SB | -0.0067 (-0.0116 to -0.0017) * | LPA | -0.0057 (-0.0104 to -0.0010) * |
| 20 |  | -0.0195 (-0.0319 to -0.0071) * |  | -0.0161 (-0.0276 to -0.0045) * |  | -0.0142 (-0.0255 to -0.0030) * |
| 30 |  | -0.0373 (-0.0631 to -0.0012) * |  | -0.0324 (-0.0562 to -0.0087) * |  | -0.0299 (-0.0541 to -0.0056) * |

*p<0.05

^a^ Adjusted for age, gender, marital status, education, household income, BMI, smoking, alcohol, chronic diseases, hours of work, and job activity

Abbreviation: BMI = body mass index, CI = confidence interval, LPA = light-intensity physical activity, min = minute, MVPA = moderate-to-vigorous-intensity physical activity, SB = sedentary behaviour
